# Association between blood heavy metal levels and subtypes of steatotic liver disease: A nationally representative cross-sectional analysis in South Korea

**DOI:** 10.1097/MD.0000000000047365

**Published:** 2026-01-23

**Authors:** Ji Hye Choi, Juyeong Kim, Yesol Yim, Hyunjee Kim, Jiyoung Hwang, Ho Geol Woo, Sang Youl Rhee, Yerin Hwang, Dong Keon Yon

**Affiliations:** a Center for Digital Health, Medical Science Research Institute, Kyung Hee University Medical Center, Kyung Hee University College of Medicine, Seoul, South Korea; b Department of Regulatory Science, Kyung Hee University, Seoul, South Korea; c Department of Precision Medicine, Kyung Hee University School of Medicine, Seoul, South Korea; d Department of Neurology, Kyung Hee University Medical Center, Kyung Hee University College of Medicine, Seoul, South Korea; e Department of Endocrinology and Metabolism, Kyung Hee University School of Medicine, Seoul, South Korea; f Department of Pediatrics, Kyung Hee University College of Medicine, Seoul, South Korea.

**Keywords:** ALD, blood heavy metal, cadmium, lead, MASLD, mercury, MetALD, South Korea

## Abstract

Most prior studies have investigated the association between heavy metals and nonalcoholic fatty liver disease as a single entity; however, there is limited evidence regarding subtype-specific associations with steatotic liver disease (SLD) as defined by the new criteria. Thus, this study investigates associations between blood levels of heavy metals and SLD subtypes using nationally representative data from South Korea. Data were obtained from the Korea National Health and Nutrition Examination Survey between 2005 and 2017, including adults aged 19 years and older. Blood levels of heavy metals, including lead (Pb), mercury (Hg), and cadmium (Cd), were examined. SLD, estimated using the hepatic steatosis index with alcohol consumption, was categorized into metabolic dysfunction–associated SLD (MASLD), metabolic alcohol-related liver disease (MetALD), and alcohol-related liver disease (ALD). Weighted logistic regression was used to assess the association between blood concentrations of Pb, Hg, and Cd and the prevalence of each SLD subtype. The associations with metabolic biomarkers were evaluated using a weighted generalized linear model, and subgroup analyses were conducted by sex. A total of 18,871 adults (9117 males [48.31%]) with available heavy metal-related data were included in the analysis. Blood Pb levels were associated with a decreased risk of MASLD (adjusted odds ratio [aOR], 0.90 [95% CI: 0.86–0.95]) but an increased risk of ALD (1.21 [1.15–1.27]). Blood Hg level was associated with MASLD (1.03 [95% CI: 1.01–1.04]), MetALD (1.07 [1.05–1.09]), and ALD (1.02 [1.01–1.03]), with a linear trend in disease risk across quartiles. Blood Cd level showed no associations overall but was significantly associated with MetALD (1.59 [95% CI: 1.11–2.27]) and ALD (1.52 [1.24–1.85]) in male only. Both blood Hg and Cd levels were positively associated with liver enzymes and metabolic parameters. This first nationally representative study using the new classification highlights the subtype-specific impact of heavy metals on SLD. The findings suggest Hg and Cd link metabolic dysfunction to liver disease, underscoring the need for subtype-tailored environmental risk assessments and precision public health strategies.

## 1. Introduction

Steatotic liver disease (SLD) has become a major global health concern and is now one of the most prevalent causes of chronic liver disease.^[1]^ With the decline of viral hepatitis due to improved antiviral treatments and vaccination programs, the burden of liver disease has increasingly shifted toward metabolic and lifestyle-related causes.^[2]^ SLD is estimated to affect nearly one-third of adults worldwide,^[3,4]^ with rising prevalence linked to the global increase in obesity, type 2 diabetes, and metabolic syndrome.^[1,5]^ Beyond hepatic complications like cirrhosis and hepatocellular carcinoma, SLD is also associated with extrahepatic conditions such as cardiovascular disease and chronic kidney disease, highlighting the need for effective prevention and management strategies.

Given its central role in detoxification, the liver is particularly susceptible to environmental toxicants, including heavy metals such as lead (Pb), mercury (Hg), and cadmium (Cd). These metals, commonly introduced through diet, smoking, and occupational exposure,^[4]^ can accumulate in the body and induce hepatotoxicity. The association between blood concentrations of Pb, Hg, and Cd and SLD subtypes is believed to stem from diverse toxicological and metabolic mechanisms.^[6]^ These heavy metals are thought to exert hepatotoxic effects through mechanisms such as oxidative stress, mitochondrial dysfunction, endocrine and metabolic dysregulation, and alterations in the gut-liver axis.^[6,7]^ Previous studies suggest that exposure to heavy metals can increase the production of reactive oxygen species (ROS), which in turn contributes to lipid peroxidation, inflammatory responses, and the activation of apoptotic signaling pathways, potentially leading to hepatocellular injury and fibrosis.^[6–8]^ Furthermore, heavy metals can deplete intracellular glutathione and impair the activity of antioxidant enzymes, thereby disrupting redox homeostasis and potentially increasing genotoxic risk.

The terminology for fatty liver disease has recently shifted from nonalcoholic fatty liver disease (NAFLD) to SLD, reflecting a broader classification based on metabolic dysfunction and alcohol intake.^[9]^ Under this framework, SLD is subdivided into metabolic dysfunction-associated SLD (MASLD), metabolic dysfunction-associated alcoholic liver disease (MetALD), and alcohol-related liver disease (ALD).^[9]^ This reclassification reflects the clinical and pathophysiological heterogeneity of the disease. However, most prior studies on heavy metals and liver disease have treated NAFLD as a single entity,^[7,9]^ limiting insight into subtype-specific effects.

Therefore, the present study aimed to comprehensively investigate the associations between blood concentrations of Pb, Hg, and Cd and the subtypes of SLD using data from the Korea National Health and Nutrition Examination Survey (KNHANES).^[10]^ By evaluating subtype-specific associations, this study seeks to advance understanding of how internal heavy metal burden contributes to liver disease development and to inform precision public health strategies tailored to both disease subtype and exposure profile.

## 2. Methods

### 2.1. Data source

This study utilized data from the KNHANES, a nationwide, population-based survey conducted by the Korea Disease Control and Prevention Agency.^[10]^ To ensure representativeness and minimize bias, KNHANES employs a stratified, multistage cluster sampling design.^[11]^ Sampling weights were applied to accurately reflect the distribution of the Korean population, thereby allowing for valid national estimates of health and nutrition indicators. The analysis included data from the years 2005 to 2017 in which heavy metal concentration measurements were available. Participants were limited to Korean adults aged 19 years or older. After excluding individuals with missing or incomplete data, a final analytic sample of 18,871 individuals was used to evaluate associations between blood concentrations of heavy metals and subtypes of SLD (MASLD, MetALD, and ALD). The study protocol was approved by the Institutional Review Board of the Korea Disease Control and Prevention Agency (2008-04EXP-01-C, 2010-02CON-21-C, 2011-02CON-06-C, 2012-01EXP-01-2C, 2013-07CON-03-4C, 2013-12EXP-03-5C, 2018-01-03-P-A, 2018-01-03-C-A, 2018-01-03-2C-A, 2018-01-03-5C-A, 2018-01-03-4C-A). Written informed consent was obtained directly by KNHANES from all participants before data collection. The data are fully anonymized prior to public release, and this study used only these de-identified secondary data. Therefore, no additional consent from participants was required. All procedures adhered to the ethical principles of the Declaration of Helsinki.

### 2.2. Assessment of heavy metal exposure

Exposure to heavy metals was assessed based on blood concentrations of Pb, Hg, and Cd, as measured in the KNHANES. Heavy metal testing was conducted using a subsample design that varied by survey year, with participants randomly selected by sex and age group within each enumeration district. Blood concentrations of Pb and Cd were measured using atomic absorption spectrophotometry with the PerkinElmer AAnalyst 600 (PerkinElmer, Turku, Finland), while Hg was analyzed using the gold amalgamation method with the DMA-80 (Milestone, Bergamo, Italy). The concentrations were recorded in µg/dL for Pb and in µg/L for Hg and Cd. When each metal was categorized into quartiles, the corresponding values for each quartile were as follows: Pb, <1.52 (Q1), 1.52–2.08 (Q2), 2.09–2.80 (Q3), >2.80 (Q4); Hg, <2.43 (Q1), 2.43–3.66 (Q2), 3.67–5.61 (Q3), >5.61 (Q4); Cd, <0.68 (Q1), 0.68–1.03 (Q2), 1.04–1.50 (Q3), >1.50 (Q4).

### 2.3. Ascertainment of steatotic liver disease

This study aimed to examine the effect of heavy metal concentrations on the risk of SLD. The diagnosis of SLD was performed in 2 stages. In the first stage, hepatic steatosis was assessed using the hepatic steatosis index (HSI), a noninvasive surrogate marker derived from body-mass index (BMI), alanine aminotransferase (ALT), and aspartate aminotransferase (AST) levels. The HSI was calculated using the following formula: HSI = 8 × (ALT/ AST) + BMI.^[12]^ An additional 3 points were added for participants with diabetes, and 2 points were added for female participants. Individuals with an HSI score of 36 or greater were considered to have a high probability of hepatic steatosis. In the second stage, individuals with suspected hepatic steatosis were further classified into 3 categories: MASLD, MetALD, and ALD, based on metabolic status and alcohol consumption.^[13]^ Metabolic dysfunction was defined as the presence of at least one of the following conditions: BMI ≥ 23 kg/m^2^; waist circumference ≥ 90 cm for male or ≥85 cm for female; fasting blood glucose ≥ 100 mg/dL; glycated hemoglobin ≥ 5.7%; a history of diabetes; blood pressure ≥ 130/85 mm Hg or current use of antihypertensive medication; high-density lipoprotein cholesterol < 40 mg/dL in male or <50 mg/dL in female; or current use of lipid-lowering medication. Participants who met at least one of these criteria and consumed alcohol less than or equal to 30 grams per day for male and 20 grams per day for female were classified as having MASLD.^[14]^ Those who met the same metabolic criteria but consumed alcohol above these thresholds were categorized as MetALD. Lastly, individuals who did not meet any of the metabolic dysfunction criteria but exceeded the alcohol consumption thresholds were classified as having ALD.

### 2.4. Covariates

The covariates included age, sex (male and female), residential area (urban and rural),^[15]^ education level (elementary school or less, middle school, high school, college or higher, and unknown),^[16]^ household income (lowest, second, third, highest quartile, and unknown), smoking status (nonsmoker, ex-smoker, current smoker, and unknown), and total caloric intake (lowest, second, third, highest quartile, and unknown). Clinical and physiological indicators directly used in disease diagnosis – such as AST, ALT, BMI, blood pressure, and fasting blood glucose – were analyzed separately to assess their associations with heavy metal exposure and were not included as covariates in the regression models.

### 2.5. Statistical analysis

Continuous variables were summarized as survey-weighted means with standard deviations, and categorical variables as survey-weighted frequencies and percentages. Group comparisons, including quartile-based comparisons (Q1–Q4) and sex-stratified analyses, were performed using survey-weighted logistic regression models for binary outcomes and survey-weighted general linear models for continuous outcomes, from which *P*-values were derived.

To assess the association between blood concentrations of heavy metals and the risk of SLD, we conducted weighted logistic regression analyses. Subtypes of SLD (MASLD, MetALD, and ALD) were used as dependent variables, with blood levels of Pb, Hg, and Cd as independent variables. The results are reported as adjusted odds ratios (aORs) with corresponding 95% confidence intervals (CIs). To further explore the relationship between heavy metal exposure and individual metabolic or hepatic markers, we employed weighted general linear models, treating each diagnostic indicator of SLD as a continuous outcome. These associations are presented as β coefficients with 95% CIs.

All models were adjusted for key sociodemographic and behavioral covariates, including region of residence, education level, household income, smoking status, and total caloric intake. Sex and age were included a priori based on their well-established roles as confounders. Additional covariates identified through univariate analysis (*P* < .10) were also included in the final multivariable models.^[17]^ To evaluate potential effect modification, sex-stratified analyses were performed. Each stratified model included all covariates except the stratifying variable itself. All statistical analyses were performed using SAS software (version 9.4; SAS Institute, Cary). A two-sided *P*-value < .05 was considered statistically significant.

## 3. Results

This study analyzed the association between blood heavy metal concentrations and MASLD, MetALD, and ALD among 18,871 adult participants from the KNHANES conducted between 2005 and 2017 (Table 1 and Table S1, Supplemental Digital Content, https://links.lww.com/MD/R220). Of the 18,871 adults included in the analysis (9117 males [48.31%]), 3603 (19.0%) were classified as having MASLD, 682 (3.6%) as MetALD, and 2637 (14.0%) as ALD. Participants with missing blood heavy metal data and those under 19 years of age were excluded from the analysis (Fig. S1, Supplemental Digital Content, https://links.lww.com/MD/R219).

**Table 1:** Crude baseline characteristics of the study population by each heavy metal quartile during the entire study period (n = 18,871).

Variables	Total	Lead				Mercury				Cadmium			
		Q1	Q2	Q3	Q4	Q1	Q2	Q3	Q4	Q1	Q2	Q3	Q4
Overall, n	18,871	4719 (25.01)	4714 (24.98)	4720 (25.01)	4718 (25.00)	4718 (25.00)	4718 (25.00)	4718 (25.00)	4717 (25.00)	4714 (24.98)	4727 (25.05)	4707 (24.94)	4723 (25.03)
Age, yr, mean (SD)	46.32 (15.41)	40.53 (15.65)	44.97 (15.67)	48.50 (14.67)	51.27 (13.43)	45.26 (17.52)	44.90 (15.64)	46.66 (14.61)	48.45 (13.34)	36.31 (14.10)	46.07 (14.85)	50.46 (14.20)	52.13 (13.58)
Sex, n (%)													
Male	9117 (48.31)	1230 (6.52)	1989 (10.54)	2598 (13.77)	3300 (17.49)	1462 (7.75)	1976 (10.47)	2461 (13.04)	3218 (17.05)	2685 (14.23)	2438 (12.92)	2131 (11.29)	1863 (9.87)
Female	9754 (51.69)	3489 (18.49)	2725 (14.44)	2122 (11.24)	1418 (7.51)	3256 (17.25)	2742 (14.53)	2257 (11.96)	1499 (7.94)	2029 (10.75)	2289 (12.13)	2576 (13.65)	2860 (15.16)
Region of residence, n (%)													
Urban	8821 (46.74)	2326 (12.33)	2263 (11.99)	2192 (11.62)	2040 (10.81)	2186 (11.58)	2241 (11.88)	2193 (11.62)	2201 (11.66)	2377 (12.60)	2222 (11.77)	2174 (11.52)	2048 (10.85)
Rural	10,050 (53.26)	2393 (12.68)	2451 (12.99)	2528 (13.40)	2678 (14.19)	2532 (13.42)	2477 (13.13)	2525 (13.38)	2516 (13.33)	2337 (12.38)	2505 (13.27)	2533 (13.42)	2675 (14.18)
Education, n (%)													
Elementary school or less	3430 (18.18)	525 (2.78)	779 (4.13)	935 (4.95)	1191 (6.31)	971 (5.15)	838 (4.44)	835 (4.42)	786 (4.17)	287 (1.52)	668 (3.54)	1046 (5.54)	1429 (7.57)
Middle school	1940 (10.28)	276 (1.46)	420 (2.23)	555 (2.94)	689 (3.65)	411 (2.18)	469 (2.49)	495 (2.62)	565 (2.99)	206 (1.09)	472 (2.50)	602 (3.19)	660 (3.50)
High school	6793 (36.00)	1793 (9.50)	1727 (9.15)	1651 (8.75)	1622 (8.60)	1792 (9.50)	1673 (8.87)	1758 (9.32)	1570 (8.32)	1819 (9.64)	1688 (8.94)	1694 (8.98)	1592 (8.44)
College or more	6181 (32.75)	1978 (10.48)	1644 (8.71)	1460 (7.74)	1099 (5.82)	1394 (7.39)	1614 (8.55)	1519 (8.05)	1654 (8.76)	2270 (12.03)	1765 (9.35)	1232 (6.53)	914 (4.84)
Unknown	527 (2.79)	147 (0.78)	144 (0.76)	119 (0.63)	117 (0.62)	150 (0.79)	124 (0.66)	111 (0.59)	142 (0.75)	132 (0.70)	134 (0.71)	133 (0.70)	128 (0.68)
Household income, n (%)													
Lowest quartile	3105 (16.45)	606 (3.21)	718 (3.80)	813 (4.31)	968 (5.13)	992 (5.26)	750 (3.97)	710 (3.76)	653 (3.46)	466 (2.47)	625 (3.31)	885 (4.69)	1129 (5.98)
Second quartile	4833 (25.61)	1174 (6.22)	1174 (6.22)	1198 (6.35)	1287 (6.82)	1307 (6.93)	1289 (6.83)	1224 (6.49)	1013 (5.37)	1122 (5.95)	1266 (6.71)	1231 (6.52)	1214 (6.43)
Third quartile	5248 (27.81)	1432 (7.59)	1340 (7.10)	1283 (6.80)	1193 (6.32)	1253 (6.64)	1361 (7.21)	1336 (7.08)	1298 (6.88)	1422 (7.54)	1392 (7.38)	1264 (6.70)	1170 (6.20)
Highest quartile	5494 (29.11)	1470 (7.79)	1443 (7.65)	1380 (7.31)	1201 (6.36)	1120 (5.94)	1282 (6.79)	1397 (7.40)	1695 (8.98)	1662 (8.81)	1396 (7.40)	1285 (6.81)	1151 (6.10)
Unknown	191 (1.01)	37 (0.20)	39 (0.21)	46 (0.24)	69 (0.37)	46 (0.24)	36 (0.19)	51 (0.27)	58 (0.31)	42 (0.22)	48 (0.25)	42 (0.22)	59 (0.31)
Smoking status, n (%)													
nonsmoker	10,334 (54.76)	3509 (18.59)	2843 (15.07)	2317 (12.28)	1665 (8.82)	3250 (17.22)	2855 (15.13)	2395 (12.69)	1834 (9.72)	2797 (14.82)	2470 (13.09)	2484 (13.16)	2583 (13.69)
Ex-smoker	3730 (19.77)	609 (3.23)	829 (4.39)	1044 (5.53)	1248 (6.61)	628 (3.33)	823 (4.36)	988 (5.24)	1291 (6.84)	1183 (6.27)	1114 (5.90)	841 (4.46)	0.592 (3.14)
Current smoker	4433 (23.49)	529 (2.80)	940 (4.98)	1265 (6.70)	1699 (9.00)	731 (3.87)	961 (5.09)	1250 (6.62)	1491 (7.90)	645 (3.42)	1063 (5.63)	1277 (6.77)	1448 (7.67)
Unknown	374 (1.98)	72 (0.38)	102 (0.54)	94 (0.50)	106 (0.56)	109 (0.58)	79 (0.42)	85 (0.45)	101 (0.54)	89 (0.47)	80 (0.42)	105 (0.56)	100 (0.53)
Total amount of kcal, n (%)													
Lowest quartile	4072 (25.00)	1242 (6.58)	1077 (5.71)	959 (5.08)	794 (4.21)	1305 (6.92)	1103 (5.84)	910 (4.82)	754 (4.00)	880 (4.66)	980 (5.19)	1070 (5.67)	1142 (6.05)
Second quartile	4072 (25.00)	1073 (5.69)	1029 (5.45)	994 (5.27)	976 (5.17)	1099 (5.82)	1055 (5.59)	984 (5.21)	934 (4.95)	863 (4.57)	990 (5.25)	1124 (5.96)	1095 (5.80)
Third quartile	4072 (25.00)	988 (5.24)	1008 (5.34)	1012 (5.36)	1064 (5.64)	1000 (5.30)	986 (5.22)	1064 (5.64)	1022 (5.42)	1048 (5.55)	1010 (5.35)	986 (5.22)	1028 (5.45)
Highest quartile	4072 (25.00)	863 (4.57)	955 (5.06)	1062 (5.63)	1192 (6.32)	776 (4.11)	977 (5.18)	1086 (5.75)	1233 (6.53)	1170 (6.20)	1096 (5.81)	950 (5.03)	856 (4.54)
Unknown	2583 (13.69)	553 (2.93)	645 (3.42)	693 (3.67)	692 (3.67)	538 (2.85)	597 (3.16)	674 (3.57)	774 (4.10)	753 (3.99)	651 (3.45)	577 (3.06)	602 (3.19)
MASLD, n (%)													
Yes	3603 (19.09)	870 (4.61)	978 (5.18)	906 (4.80)	849 (4.50)	785 (4.16)	809 (4.29)	993 (5.26)	1016 (5.38)	858 (4.55)	860 (4.56)	914 (4.84)	971 (5.15)
No	15,268 (80.91)	3849 (20.40)	3736 (19.80)	3814 (20.21)	3869 (20.50)	3933 (20.84)	3909 (20.71)	3725 (19.74)	3701 (19.61)	3856 (20.43)	3867 (20.49)	3793 (20.10)	3752 (19.88)
MetALD, n (%)													
Yes	682 (3.61)	106 (0.56)	142 (0.75)	220 (1.17)	214 (1.13)	62 (0.33)	154 (0.82)	199 (1.05)	267 (1.41)	149 (0.79)	192 (1.02)	183 (0.97)	158 (0.84)
No	18,189 (96.39)	4613 (24.44)	4572 (24.23)	4500 (23.85)	4504 (23.87)	4656 (24.67)	4564 (24.19)	4519 (23.95)	4450 (23.58)	4565 (24.19)	4535 (24.03)	4524 (23.97)	4565 (24.19)
ALD, n (%)													
Yes	2637 (13.97)	434 (2.30)	554 (2.94)	688 (3.65)	961 (5.09)	507 (2.69)	607 (3.22)	658 (3.49)	865 (4.58)	634 (3.36)	668 (3.54)	694 (3.68)	641 (3.40)
No	16,234 (86.03)	4285 (22.71)	4160 (22.04)	4032 (21.37)	3757 (19.91)	4211 (22.31)	4111 (21.78)	4060 (21.51)	3852 (20.41)	4080 (21.62)	4059 (21.51)	4013 (21.27)	4082 (21.63)

ALD = alcohol-related liver disease, MASLD = metabolic dysfunction-associated steatotic liver disease, MetALD = metabolic alcohol-related liver disease, SD = standard deviation.

**Figure 1.**
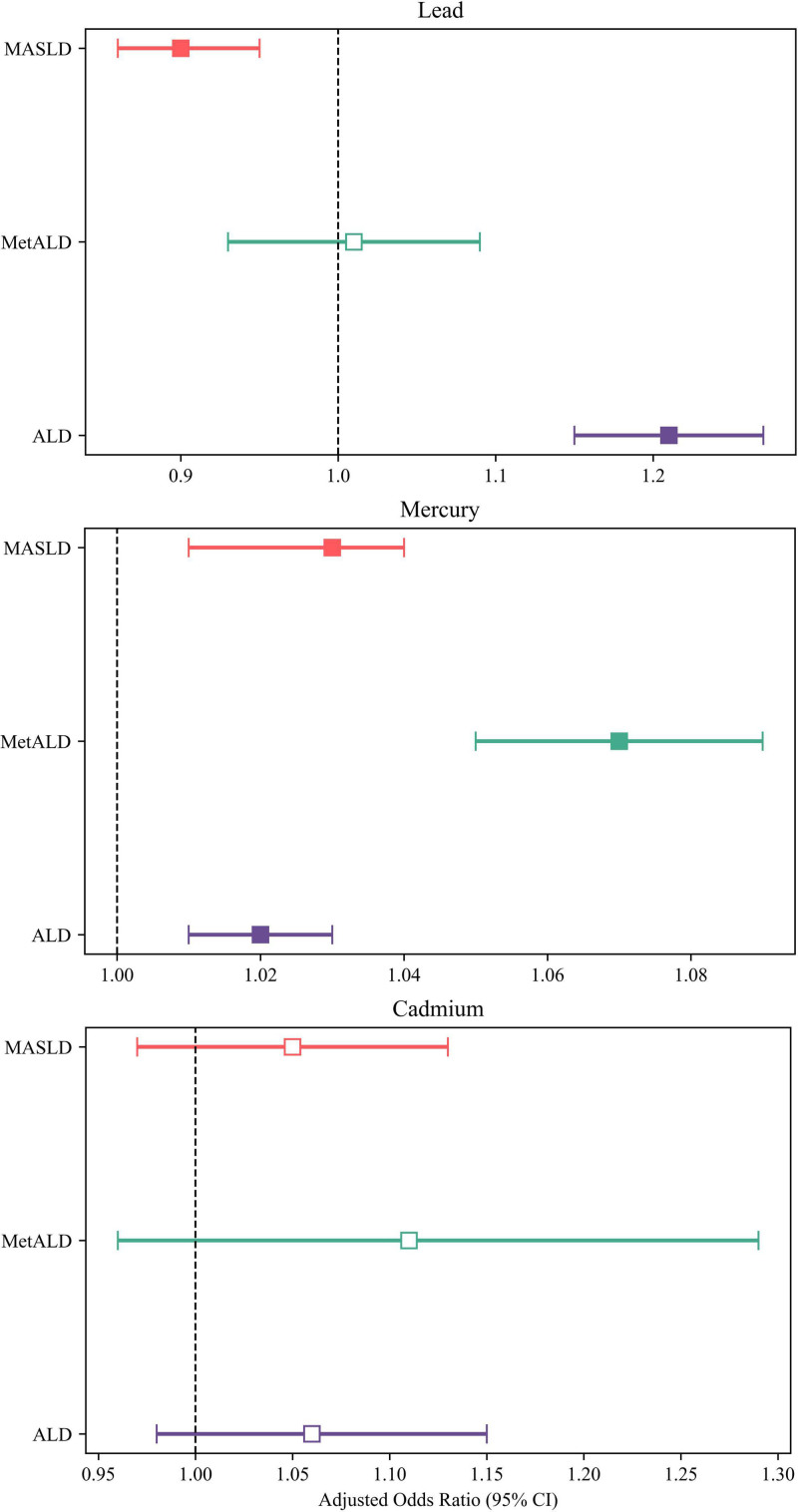
Adjusted odds ratios of MASLD, MetALD, and ALD in relation to blood levels of lead, mercury, and cadmium. ALD = alcohol-related liver disease, CI = confidence interval, MASLD = metabolic dysfunction-associated steatotic liver disease, MetALD = metabolic alcohol-related liver disease.

In the analysis, blood Pb levels were inversely associated with MASLD (aOR, 0.90 [95% CI: 0.86–0.95]) and positively associated with ALD (1.21 [1.15–1.27]; Fig. 1 and Table 2). In contrast, blood Hg levels showed positive associations with all subtypes of SLD – MASLD (1.03 [95% CI: 1.01–1.04]), MetALD (1.07 [1.05–1.09]), and ALD (1.02 [1.01–1.03]). Blood Cd levels were not significantly associated with any subtypes of SLD. However, in the generalized linear model, Cd levels showed positive associations with AST (adjusted β, 2.44 [95% CI: 2.09–2.78]), ALT (2.92 [2.47–3.37]), and triglycerides (TG; 6.29 [3.61–8.98]), and a negative association with fasting blood glucose (−0.81 [−1.32 to −0.31]; Table 2). Blood Hg levels were positively associated with multiple metabolic biomarkers, including AST (0.16 [95% CI: 0.10–0.22]), ALT (0.30 [0.22–0.38]), and fasting blood glucose (0.16 [0.08–0.25]), suggesting a potential adverse impact on metabolic health. Blood Pb levels were negatively associated with ALT (95% CI: −0.41 [-0.66 to −0.16]), BMI (−0.06 [−0.11 to −0.02]), waist circumference (−0.22 [−0.34 to −0.11]), and fasting blood glucose (−0.49 [−0.77 to −0.21]), while showing positive associations with HDL cholesterol (0.44 [0.27–0.62]) and TG (1.92 [0.44–3.41]), indicating a mixed metabolic profile.

**Table 2:** Multivariable analysis of the relationship of blood levels of 3 heavy metals (continuous variables) with MASLD, MetALD, ALD, and HSI index components among KNHANES participants who completed the questionnaire and blood test.

Variables	Independent variables					
	Lead		Mercury		Cadmium	
	aOR (95% CI)	*P* value	aOR (95% CI)	*P* value	aOR (95% CI)	*P* value
Binary logistic regression analysis						
MASLD*	**0.90 (0.86–0.95**)	**<.001**	**1.03 (1.01–1.04**)	**<.001**	1.05 (0.97–1.13)	.24
MetALD†	1.01 (0.93–1.09)	.84	**1.07 (1.05–1.09**)	**<.0001**	1.11 (0.96–1.29)	.16
ALD‡	**1.21 (1.15–1.27**)	**<.0001**	**1.02 (1.01–1.03**)	**<.01**	1.06 (0.98–1.15)	.15

	aβ (95% CI)	*P* value	aβ (95% CI)	*P* value	aβ (95% CI)	*P* value
General linear model analysis						
AST	0.06 (-0.13 to 0.25)	.56	**0.16 (0.10–0.22**)	**<.0001**	**2.44 (2.09–2.78**)	**<.0001**
ALT	**-0.41 (-0.66 to −0.16**)	**<.01**	**0.30 (0.22–0.38**)	**<.0001**	**2.92 (2.47–3.37**)	**<.0001**
BMI	**-0.06 (-0.11 to −0.02**)	**<.01**	**0.09 (0.08–0.11**)	**<.0001**	**0.12 (0.04–0.20**)	**<.01**
Waist circumference	**-0.22 (-0.34 to −0.11**)	**<.001**	**0.27 (0.23–0.30**)	**<.0001**	**0.38 (0.17–0.59**)	**<.001**
Fasting blood glucose	**-0.49 (-0.77 to −0.21**)	**<.001**	**0.16 (0.08–0.25**)	**<.001**	**-0.81 (-1.32 to −0.31**)	**<.01**
HbA1c	0.01 (-0.01 to 0.03)	.32	**0.01 (0.00–0.01**)	**<.01**	0.00 (-0.03 to 0.03)	.91
SBP	**0.51 (0.32–0.70**)	**<.0001**	**0.13 (0.07–0.19**)	**<.0001**	**1.66 (1.32–2.01**)	**<.0001**
DBP	**0.65 (0.52–0.78**)	**<.0001**	**0.18 (0.14–0.22**)	**<.0001**	**1.53 (1.30–1.76**)	**<.0001**
HDL-C	**0.44 (0.27–0.62**)	**<.0001**	0.01 (-0.04 to 0.06)	0.70	0.03 (-0.27 to 0.34)	.84
TG	**1.92 (0.44–3.41**)	.01	**1.17 (0.71–1.64**)	**<.0001**	**6.29 (3.61–8.98**)	**<.0001**

The numbers in bold indicate statistical significance (*P*-value <.05).

ALD = alcohol-related liver disease, ALT = alanine aminotransferase, AST = aspartate aminotransferase, BMI = body-mass index, CI = confidence interval, DBP = diastolic blood pressure, HbA1c = glycated hemoglobin, HDL-C = high-density lipoprotein cholesterol, HSI = hepatic steatosis index, MASLD = metabolic dysfunction-associated steatotic liver disease, MetALD = metabolic alcohol-related liver disease, OR = odds ratio, SBP = systolic blood pressure, TG = triglyceride.

* The logistic regression model was adjusted for sex (male and female), age, education level (elementary school or less, middle school, high school, college or more, and unknown), household income (lowest, second, third, highest quartile, and unknown), smoking status (nonsmoker, ex-smoker, current smoker and unknown) and total caloric intake (lowest, second, third, highest quartile, and unknown).

† The logistic regression model was adjusted for sex, age, region of residence, education level, household income, smoking status and total caloric intake.

‡ The logistic regression model was adjusted for sex, age, household income, smoking status and total caloric intake.

**Figure 2.**
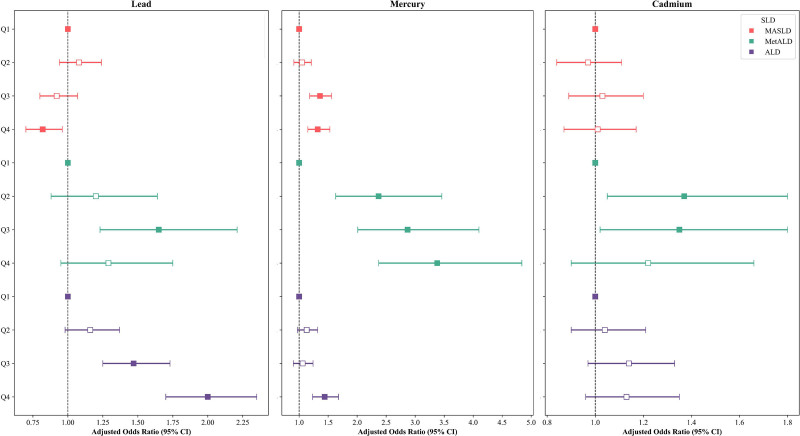
Adjusted odds ratios for MASLD, MetALD, and ALD across quartiles of blood lead, mercury, and cadmium levels. ALD = alcohol-related liver disease, CI = confidence interval, MASLD = metabolic dysfunction-associated steatotic liver disease, MetALD = metabolic alcohol-related liver disease, SLD = steatotic liver disease.

Similar trends were observed when heavy metal concentrations were categorized into quartiles (Fig. 2 and Table 3). Blood Pb levels exhibited an inverse association with MASLD, showing a significantly lower aOR in the highest quartile (Q4; aOR, 0.82 [95% CI: 0.70–0.96]). In contrast, for ALD, the risk increased progressively with higher concentrations (Q3: 1.47 [95% CI: 1.25–1.73]; Q4: 2.00 [1.70–2.35]). Blood Hg levels showed a positive association with all subtypes of SLD, with increasing odds observed across exposure quartiles. For both MetALD and ALD, individuals in Q4 had significantly higher odds of the disease (MASLD: 1.32 [95% CI: 1.15–1.53]; ALD: 1.44 [1.23–1.68]). A linear increase in the odds of having MetALD was observed across quartiles of blood Hg concentration: Q2, 2.37 (95% CI: 1.63–3.46); Q3, 2.87 (2.01–4.10); Q4, 3.38 (2.37–4.84).

**Table 3:** Multivariable analysis of the association between quartiles of blood levels of 3 heavy metals and MASLD, MetALD, and ALD among KNHANES participants.

Variables	MASLD		MetALD		ALD	
	Model 1*	Model 2†	Model 1*	Model 2‡	Model 1*	Model 2§
Lead						
Q1	1.00 (ref)	1.00 (ref)	1.00 (ref)	1.00 (ref)	1.00 (ref)	1.00 (ref)
Q2	**1.16 (1.02–1.33**)	1.08 (0.94–1.24)	1.05 (0.90–1.21)	1.05 (0.91–1.21)	1.00 (0.88–1.15)	0.97 (0.84–1.11)
Q3	1.05 (0.92–1.20)	0.92 (0.80–1.07)	**1.37 (1.19–1.57**)	**1.36 (1.18–1.56**)	1.09 (0.95–1.25)	1.03 (0.89–1.20)
Q4	0.99 (0.86–1.14)	**0.82 (0.70–0.96**)	**1.36 (1.18–1.56**)	**1.32 (1.15–1.53**)	1.08 (0.95–1.23)	1.01 (0.87–1.17)
Mercury						
Q1	1.00 (ref)	1.00 (ref)	1.00 (ref)	1.00 (ref)	1.00 (ref)	1.00 (ref)
Q2	**1.54 (1.15–2.06**)	1.20 (0.88–1.64)	**2.84 (1.96–4.12**)	**2.37 (1.63–3.46**)	**1.40 (1.09–1.80**)	**1.37 (1.05–1.80**)
Q3	**2.40 (1.82–3.16**)	**1.65 (1.23–2.21**)	**3.90 (2.75–5.54**)	**2.87 (2.01–4.10**)	**1.31 (1.02–1.69**)	**1.35 (1.02–1.80**)
Q4	**2.12 (1.60–2.80**)	1.29 (0.95–1.75)	**5.25 (3.71–7.42**)	**3.38 (2.37–4.84**)	1.09 (0.83–1.43)	1.22 (0.90–1.66)
Cadmium						
Q1	1.00 (ref)	1.00 (ref)	1.00 (ref)	1.00 (ref)	1.00 (ref)	1.00 (ref)
Q2	**1.35 (1.15–1.59**)	1.16 (0.98–1.37)	**1.31 (1.12–1.52**)	1.13 (0.97–1.32)	1.13 (0.98–1.29)	1.04 (0.90–1.21)
Q3	**1.88 (1.61–2.18**)	**1.47 (1.25–1.73**)	**1.40 (1.21–1.62**)	1.06 (0.90–1.24)	**1.21 (1.06–1.38**)	1.14 (0.97–1.33)
Q4	**2.73 (2.36–3.16**)	**2.00 (1.70–2.35**)	**2.10 (1.82–2.42**)	**1.44 (1.23–1.68**)	**1.16 (1.00–1.33**)	1.13 (0.96–1.35)

The numbers in bold indicate statistical significance (*P*-value <.05).

ALD = alcohol-related liver disease, CI = confidence interval, MASLD = metabolic dysfunction-associated steatotic liver disease, MetALD = metabolic alcohol-related liver disease, OR = odds ratio, SD = standard deviation.

* The model was not adjusted.

† The logistic regression model was adjusted for sex (male and female), age, education level (elementary school or less, middle school, high school, college or more, and unknown), household income (lowest, second, third, highest quartile, and unknown), smoking status (nonsmoker, ex-smoker, current smoker and unknown) and total caloric intake (lowest, second, third, highest quartile, and unknown).

‡ The logistic regression model was adjusted for sex, age, region of residence, education level, household income, smoking status and total caloric intake.

§ The logistic regression model was adjusted for sex, age, household income, smoking status and total caloric intake.

According to sex-stratified analyses, blood heavy metal concentrations were generally higher in males, who also tended to exhibit a more pronounced prevalence and risk of all subtypes of SLD (Table S2, Supplemental Digital Content, https://links.lww.com/MD/R220). However, consistent with previous findings, blood Pb levels showed contrasting associations with MASLD in both sexes, whereas the odds of ALD increased progressively across quartiles of blood Pb concentration. Blood Hg levels were positively associated with all SLD subtypes in both sexes. For MASLD, elevated risk was observed in both males (Q4: 1.30 [95% CI: 1.06–1.61]) and females (Q4: 1.28 [1.04–1.57]). Notably, MetALD showed a marked association with blood Hg levels in males (Q4: 4.03 [95% CI: 2.46–6.60]) and females (Q4: 2.60 [1.48–4.55]). ALD risk also increased in both males (Q4: 1.52 [95% CI: 1.24–1.85]) and females (Q4: 1.39 [1.09–1.78]). Blood Cd levels were significantly associated with SLD only in males, showing a trend of increasing risk with higher concentrations.

## 4. Discussion

### 4.1. Key finding

In this study, data from the KNHANES between 2005 and 2017 were used to analyze the association between blood heavy metal concentrations and subtypes of SLD. Blood Pb levels were associated with a decreased risk of MASLD but an increased risk of ALD. Blood Hg levels were associated with all subtypes of SLD, with a linear trend in disease risk across quartiles. Blood Cd level showed no associations overall but was significantly associated with MetALD and ALD in males only. Additionally, both blood Hg and Cd levels were positively associated with liver enzymes (AST and ALT) and metabolic parameters (BMI, waist circumference, systolic blood pressure, diastolic blood pressure, and TG). These findings suggest that different heavy metals may affect liver and metabolic health through distinct biological mechanisms.

### 4.2. Plausible underlying mechanisms

While the underlying mechanisms between heavy metal concentrations and SLD are complex, a primary pathway is thought to involve the induction of severe oxidative stress and disruption of metabolic homeostasis.^[7,18]^ Specifically, heavy metals such as Hg and Cd exhibit a high affinity for sulfhydryl (–SH) groups. This binding leads to the critical depletion of intracellular glutathione, the liver’s primary antioxidant defender. The resulting redox imbalance generates a surge in ROS that overwhelms the liver’s remaining antioxidant capacity.^[7]^ These ROS, in turn, trigger lipid peroxidation—a destructive chain reaction that damages hepatocyte and mitochondrial membranes. This mitochondrial damage is particularly relevant to SLD, as it can impair fatty acid β-oxidation (the breakdown of fat) and promote the “excessive accumulation of fat in the liver,” the hallmark of steatosis.^[7,18]^ Moreover, this persistent oxidative stress and lipotoxicity can activate pro-inflammatory pathways, potentially driving the progression from simple steatosis to inflammation and fibrosis.^[7]^

Although both male and females are susceptible to the health effects of heavy metal exposure; however, the patterns and magnitudes of these effects may vary by sex.^[19]^ These differences may be attributed to sex-specific physiological factors, including body composition, hormone levels, and variations in the absorption, distribution, metabolism, and excretion of heavy metals.^[19]^ Females generally have a higher percentage of body fat, which may facilitate greater accumulation of metals in the body, and sex hormones such as estrogen and testosterone are known to influence the metabolism and detoxification of certain heavy metals in distinct ways. Behavioral differences and differential exposure pathways may also contribute to sex-specific health outcomes.

### 4.3. Comparison of previous studies

Previous research findings have been inconsistent. Some epidemiological studies have reported that blood Pb exposure is associated with an increased prevalence of NAFLD and a higher risk of hepatic fibrosis,^[20]^ while a National Health and Nutrition Examination Survey (NHANES)-based study identified Pb as an independent risk factor for metabolic associated fatty liver disease (MAFLD).^[21]^ In contrast, other studies using Korean National Environmental Health Survey and NHANES data found no significant association between blood Pb levels and NAFLD.^[22]^ These conflicting results may be attributable to differences in study populations, diagnostic criteria (NAFLD vs MAFLD vs MASLD), and levels of Pb exposure. Moreover, many prior studies were limited by relatively small sample sizes (n = 1486^[20]^ or n = 3837^[22]^ vs n = 18,871 in the present study) or did not simultaneously account for the different subtypes of SLD, thereby limiting their ability to comprehensively assess the impact of Pb on liver disease.

Previous studies have reported associations between blood Hg levels and hepatic steatosis; however, their findings were somewhat limited by the study population and the scope of metabolic indicators analyzed. For example, a study based on Korean National Environmental Health Survey data reported that higher blood Hg levels were associated with increased NAFLD risk not only in overweight individuals but also in those with normal weight.^[23]^ Another study found that Hg levels significantly predicted the risk of hepatic steatosis in both sexes and showed a positive association with elevated ALT levels.^[24,25]^ However, these studies were often restricted to elderly populations^[26]^ or focused on a limited set of metabolic biomarkers (e.g., systolic blood pressure, BMI, fasting blood glucose, and TG).^[24]^ In contrast, the present study included the entire adult population aged 19 years and older and conducted a comprehensive analysis that encompassed a broader range of metabolic indicators beyond disease prevalence, including liver enzyme levels, TG, waist circumference, and high-density lipoprotein cholesterol. Our findings showed that blood Hg levels were significantly associated with all 3 subtypes of SLD and exhibited positive associations with various metabolic indicators, suggesting a potential link between Hg exposure and broader metabolic dysfunction.

Sex-stratified analysis revealed significant associations between Cd and both MetALD and ALD in male only, partially aligning with existing literature. For example, KNHANES-based studies have shown that elevated blood Cd levels are significantly associated with increased ALT, gamma-glutamyl transferase, fatty liver index, and HSI.^[27]^ Another study found that urinary Cd concentrations were positively associated with HSI and NAFLD risk.^[22]^ On the other hand, one study reported that while blood Cd levels was significantly related to HSI and fibrosis-4 index in females, only the association with fibrosis-4 remained significant after multivariable adjustment.^[24]^ These findings support the results of the present study showing no significant association between Cd and subtypes of SLD in females. In addition, the observed sex differences may be influenced by a variety of factors, including lifestyle behaviors, exposure routes, sex hormone levels, and physiological or metabolic differences in the processing of heavy metals.^[19]^ For example, smoking, occupational exposure to Cd, and the regulatory effects of sex hormones such as testosterone and estrogen on metal metabolism may partially explain the differential associations observed between males and females. These factors should be taken into account when interpreting sex-stratified analyses.

### 4.4. Strengths and limitations

This study has several limitations. First, the cross-sectional design precludes the establishment of a clear causal relationship between heavy metal concentrations and SLD subtypes. Second, blood concentrations reflect recent exposure and may not adequately represent the cumulative body burden from long-term exposure. Future studies should consider incorporating biomarkers of chronic accumulation, such as those in urine or hair. Third, SLD classification was based on the HSI, a surrogate biochemical marker, rather than on histological or imaging-based diagnosis. As HSI has limited sensitivity and specificity, particularly in Korean populations,^[28]^ there is a potential risk of misclassification. Fourth, we could not account for all potential sources of heavy metal exposure (e.g., specific dietary components, drinking water, or air quality), leaving a possibility of residual confounding. We did, however, adjust for a wide range of lifestyle and demographic factors. Lastly, the use of data from a single country (South Korea) may limit the generalizability of our findings to other ethnicities or environments.

Despite these limitations, the present study has several noteworthy strengths. Unlike many previous studies that relied on single diagnostic entity (e.g., NAFLD or MAFLD), this study adopted the newly proposed international classification of SLD, distinguishing MASLD, MetALD, and ALD. This approach allowed for a more detailed evaluation of disease-specific pathophysiology and the heterogeneous effects of heavy metal exposure. By adopting this classification framework, our study provides a robust foundation for developing targeted prevention and intervention strategies tailored to the etiological subtypes of liver disease.

### 4.5. Policy implications

The level of heavy metal exposure varies significantly depending on a country’s degree of industrialization, the stringency of environmental regulations, and the dietary and lifestyle habits of its population. In South Korea, the accumulation of heavy metals such as Pb, Hg and Cd in adults primarily results from dietary intake – particularly through the consumption of seafood, rice, and vegetables.^[29]^ In addition, inhalation of airborne particulate matter containing heavy metals is an increasingly serious environmental health concern in urban areas. Notably, atmospheric concentrations of Pb and Cd have been reported to be higher in areas near industrial complexes compared to general residential zones.^[29]^ Furthermore, the finding that Cd was significantly associated with a specific disease type only in men underscores the importance of considering sex-related differences in susceptibility to heavy metal exposure when identifying high-risk groups and developing targeted public health strategies. Correspondingly, to mitigate heavy metal exposure effectively, a multifaceted approach is essential. First, it is necessary to strengthen the regulatory limits for heavy metal residues in food and to establish a robust, ongoing food monitoring system. Second, emission sources of air pollutants must be tightly controlled, and ambient air quality standards should be reinforced. Third, individuals at high risk—such as workers in metal-related industries and residents living near industrial facilities—should undergo regular biological monitoring and be included in targeted intervention programs. Particularly in regions with a high risk of exposure due to industrial activities, policy actions should be guided by targeted health impact assessments and data-driven exposure indicators.

## 5. Conclusion

This study provides a comprehensive assessment of the associations between blood concentrations of Pb, Hg, and Cd and newly defined subtypes of SLD (MASLD, MetALD, and ALD). The findings suggest that heavy metals may contribute to liver and metabolic disorders through distinct pathophysiological pathways and underscore the need for targeted preventive strategies based on specific toxicants and disease subtypes. Future research should employ prospective study designs to validate these associations and utilize multi-omics approaches to elucidate underlying biological mechanisms, ultimately supporting the development of effective public health interventions.

## Author contributions

**Conceptualization:** Ji Hye Choi, Juyeong Kim, Yerin Hwang, Dong Keon Yon.

**Data curation:** Dong Keon Yon.

**Formal analysis:** Ji Hye Choi, Juyeong Kim, Yerin Hwang, Dong Keon Yon.

**Funding acquisition:** Dong Keon Yon.

**Project administration:** Dong Keon Yon.

**Supervision:** Dong Keon Yon.

**Visualization:** Ji Hye Choi, Juyeong Kim, Yerin Hwang.

**Writing – original draft:** Ji Hye Choi, Juyeong Kim, Yerin Hwang, Dong Keon Yon.

**Writing – review & editing:** Ji Hye Choi, Juyeong Kim, Yesol Yim, Hyunjee Kim, Jiyoung Hwang, Ho Geol Woo, Sang Youl Rhee, Yerin Hwang, Dong Keon Yon.

## Supplementary Material




