# Supplementary material for: Association between blood heavy metal levels and subtypes of steatotic liver disease: A nationally representative cross-sectional analysis in South Korea
Source: Medicine (Baltimore). 2026 Jan 23;105(4):e47365. doi: 10.1097/MD.0000000000047365 (PMC12851683; doi:10.1097/MD.0000000000047365)
Supplement: Supplementary file 1 [file medi-105-e47365-s001.docx]

**Table S1.** Crude baseline characteristics of the study population by year.

| Variables | Total | Year | | | | | | | | |
| --- | --- | --- | --- | --- | --- | --- | --- | --- | --- | --- |
|  |  | 2005 | 2008 | 2009 | 2010 | 2011 | 2012 | 2013 | 2016 | 2017 |
| Overall, n | 18,871 | 1,998 (10.59) | 2,006 (10.63) | 1,991 (10.55) | 1,989 (10.54) | 2,014 (10.67) | 2,047 (10.85) | 1,995 (10.57) | 2,867 (15.19) | 1,964 (10.41) |
| Age, years, mean (SD) | 46.32 (15.41) | 45.28 (13.61) | 45.50 (15.53) | 45.47 (15.51) | 44.87 (14.79) | 44.84 (14.64) | 44.77 (14.88) | 44.35 (14.69) | 51.17 (16.67) | 48.57 (15.99) |
| Sex, n (%) |  |  |  |  |  |  |  |  |  |  |
| Male | 9,117 (48.31) | 1,000 (5.30) | 1,000 (5.30) | 996 (5.28) | 978 (5.18) | 993 (5.26) | 1,005 (5.33) | 996 (5.28) | 1,241 (6.58) | 908 (4.81) |
| Female | 9,754 (51.69) | 998 (5.29) | 1,006 (5.33) | 995 (5.27) | 1,011 (5.36) | 1,021 (5.41) | 1,042 (5.52) | 999 (5.29) | 1,626 (8.62) | 1,056 (5.60) |
| Region of residence, n (%) |  |  |  |  |  |  |  |  |  |  |
| Urban | 8,821 (46.74) | 806 (4.27) | 910 (4.82) | 928 (4.92) | 943 (5.00) | 995 (5.27) | 1,005 (5.33) | 947 (5.02) | 1,358 (7.20) | 929 (4.92) |
| Rural | 1050 (53.26) | 1,192 (6.32) | 1,096 (5.81) | 1,063 (5.63) | 1,046 (5.54) | 1,019 (5.40) | 1,042 (5.52) | 1,048 (5.55) | 1,509 (8.00) | 1,035 (5.48) |
| Education, n (%) |  |  |  |  |  |  |  |  |  |  |
| Elementary school or less | 3,430 (18.18) | 404 (2.14) | 455 (2.41) | 419 (2.22) | 359 (1.90) | 310 (1.64) | 308 (1.63) | 300 (1.59) | 592 (3.14) | 283 (1.50) |
| Middle school | 1,940 (10.28) | 229 (1.21) | 242 (1.28) | 225 (1.19) | 204 (1.08) | 210 (1.11) | 198 (1.05) | 171 (0.91) | 284 (1.51) | 177 (0.94) |
| High school | 6,793 (36.00) | 807 (4.28) | 751 (3.98) | 748 (3.96) | 722 (3.83) | 757 (4.01) | 758 (4.02) | 746 (3.95) | 867 (4.59) | 637 (3.38) |
| College or more | 6,181 (32.75) | 542 (2.87) | 553 (2.93) | 587 (3.11) | 683 (3.62) | 700 (3.71) | 698 (3.70) | 674 (3.57) | 986 (5.22) | 758 (4.02) |
| Unknown | 527 (2.79) | 16 (0.08) | 5 (0.03) | 12 (0.06) | 21 (0.11) | 37 (0.20) | 85 (0.45) | 104 (0.55) | 138 (0.73) | 109 (0.58) |
| Household income, n (%) |  |  |  |  |  |  |  |  |  |  |
| Lowest quartile | 3,105 (16.45) | 403 (2.14) | 349 (1.85) | 352 (1.87) | 322 (1.71) | 261 (1.38) | 270 (1.43) | 295 (1.56) | 550 (2.91) | 303 (1.61) |
| Second quartile | 4,833 (25.61) | 500 (2.65) | 535 (2.84) | 472 (2.50) | 531 (2.81) | 554 (2.94) | 509 (2.70) | 541 (2.87) | 709 (3.76) | 482 (2.55) |
| Third quartile | 5,248 (27.81) | 522 (2.77) | 529 (2.80) | 575 (3.05) | 552 (2.93) | 591 (3.13) | 596 (3.16) | 554 (2.94) | 764 (4.05) | 565 (2.99) |
| Highest quartile | 5,494 (29.11) | 551 (2.92) | 538 (2.85) | 576 (3.05) | 562 (2.98) | 584 (3.09) | 643 (3.41) | 597 (3.16) | 832 (4.41) | 611 (3.24) |
| Unknown | 191 (1.01) | 22 (0.12) | 55 (0.29) | 16 (0.08) | 22 (0.12) | 24 (0.13) | 29 (0.15) | 8 (0.04) | 12 (0.06) | 3 (0.02) |
| Smoking status, n (%) |  |  |  |  |  |  |  |  |  |  |
| Non-smoker | 1334 (54.76) | 1,026 (5.44) | 1,062 (5.63) | 1,081 (5.73) | 1,058 (5.61) | 1,075 (5.70) | 1,099 (5.82) | 1,080 (5.72) | 1,719 (9.11) | 1,134 (6.01) |
| Ex-smoker | 3,730 (19.77) | 386 (2.05) | 425 (2.25) | 388 (2.06) | 404 (2.14) | 414 (2.19) | 386 (2.05) | 344 (1.82) | 571 (3.03) | 412 (2.18) |
| Current smoker | 4,433 (23.49) | 530 (2.81) | 516 (2.73) | 519 (2.75) | 514 (2.72) | 489 (2.59) | 478 (2.53) | 464 (2.46) | 526 (2.79) | 397 (2.10) |
| Unknown | 374 (1.98) | 56 (0.30) | 3 (0.02) | 3 (0.02) | 13 (0.07) | 36 (0.19) | 84 (0.45) | 107 (0.57) | 51 (0.27) | 21 (0.11) |
| Total amount of kcal, n (%) |  |  |  |  |  |  |  |  |  |  |
| Lowest quartile | 4,072 (25.00) | 348 (1.84) | 492 (2.61) | 506 (2.68) | 372 (1.97) | 380 (2.01) | 418 (2.22) | 393 (2.08) | 668 (3.54) | 495 (2.62) |
| Second quartile | 4,072 (25.00) | 461 (2.44) | 484 (2.56) | 446 (2.36) | 394 (2.09) | 433 (2.29) | 409 (2.17) | 424 (2.25) | 605 (3.21) | 416 (2.20) |
| Third quartile | 4,072 (25.00) | 472 (2.50) | 411 (2.18) | 410 (2.17) | 453 (2.40) | 461 (2.44) | 446 (2.36) | 472 (2.50) | 539 (2.86) | 408 (2.16) |
| Highest quartile | 4,072 (25.00) | 464 (2.46) | 345 (1.83) | 376 (1.99) | 479 (2.54) | 487 (2.58) | 474 (2.51) | 468 (2.48) | 588 (3.12) | 391 (2.07) |
| Unknown | 2,583 (13.69) | 253 (1.34) | 274 (1.45) | 253 (1.34) | 291 (1.54) | 253 (1.34) | 300 (1.59) | 238 (1.26) | 467 (2.47) | 254 (1.35) |
| MASLD, n (%) |  |  |  |  |  |  |  |  |  |  |
| Yes | 3,603 (19.09) | 361 (1.91) | 378 (2.00) | 391 (2.07) | 351 (1.86) | 355 (1.88) | 379 (2.01) | 382 (2.02) | 620 (3.29) | 386 (2.05) |
| No | 15,268 (80.91) | 1,637 (8.67) | 1,628 (8.63) | 1,600 (8.48) | 1,638 (8.68) | 1,659 (8.79) | 1,668 (8.84) | 1,613 (8.55) | 2,247 (11.91) | 1,578 (8.36) |
| MetALD, n (%) |  |  |  |  |  |  |  |  |  |  |
| Yes | 682 (3.61) | 66 (0.35) | 69 (0.37) | 67 (0.36) | 56 (0.30) | 81 (0.43) | 73 (0.39) | 84 (0.45) | 100 (0.53) | 86 (0.46) |
| No | 18,189 (96.39) | 1,932 (10.24) | 1,937 (10.26) | 1,924 (10.20) | 1,933 (10.24) | 1,933 (10.24) | 1,974 (10.46) | 1,911 (10.13) | 2,767 (14.66) | 1,878 (9.95) |
| ALD, n (%) |  |  |  |  |  |  |  |  |  |  |
| Yes | 2,637 (13.97) | 233 (1.23) | 229 (1.21) | 260 (1.38) | 307 (1.63) | 296 (1.57) | 303 (1.61) | 352 (1.87) | 376 (1.99) | 281 (1.49) |
| No | 16,234 (86.03) | 1,765 (9.35) | 1,777 (9.42) | 1,731 (9.17) | 1,682 (8.91) | 1,718 (9.10) | 1,744 (9.24) | 1,643 (8.71) | 2,491 (13.20) | 1,683 (8.92) |

Abbreviations: SD, standard deviation; MASLD, metabolic dysfunction-associated steatotic liver disease; MetALD, metabolic alcohol-related liver disease; ALD, alcohol-related liver disease.

**Table S2.** Sex-specific multivariable analysis of the association between quartiles of blood levels of three heavy metals and MASLD, MetALD, and ALD among KNHANES participants.

| Variables | Quartile | | | |
| --- | --- | --- | --- | --- |
|  | Q1 | Q2 | Q3 | Q4 |
| **Lead** |  |  |  |  |
| **Male** |  |  |  |  |
| MASLD ^a^ |  |  |  |  |
| Mean (SD) | 1.24 (0.23) | 1.81 (0.16) | 2.43 (0.21) | 3.83 (1.37) |
| Adjusted OR (95% CI) | 1.00 (ref) | 1.01 (0.81 to 1.26) | 0.85 (0.69 to 1.05) | **0.80 (0.64 to 0.99)** |
| MetALD ^b^ |  |  |  |  |
| Mean (SD) | 1.21 (0.28) | 1.82 (0.15) | 2.45 (0.23) | 3.91 (1.83) |
| Adjusted OR (95% CI) | 1.00 (ref) | 1.08 (0.73 to 1.59) | **1.60 (1.12 to 2.27)** | 1.40 (0.98 to 1.98) |
| ALD ^c^ |  |  |  |  |
| Mean (SD) | 1.26 (0.22) | 1.81 (0.16) | 2.45 (0.21) | 4.03 (1.40) |
| Adjusted OR (95% CI) | 1.00 (ref) | **1.32 (1.02 to 1.72)** | **1.84 (1.43 to 2.36)** | **2.52 (1.98 to 3.20)** |
| **Female** |  |  |  |  |
| MASLD ^a^ |  |  |  |  |
| Mean (SD) | 1.18 (0.24) | 1.79 (0.16) | 2.39 (0.21) | 3.66 (1.08) |
| Adjusted OR (95% CI) | 1.00 (ref) | 1.01 (0.85 to 1.20) | 0.89 (0.73 to 1.09) | 0.92 (0.75 to 1.14) |
| MetALD ^b^ |  |  |  |  |
| Mean (SD) | 1.18 (0.26) | 1.75 (0.14) | 2.43 (0.21) | 3.51 (0.45) |
| Adjusted OR (95% CI) | 1 (Reference) | **1.70 (1.05 to 2.75)** | **2.09 (1.34 to 3.24)** | 1.31 (0.77 to 2.22) |
| ALD ^c^ |  |  |  |  |
| Mean (SD) | 1.20 (0.25) | 1.78 (0.17) | 2.40 (0.20) | 3.59 (1.05) |
| Adjusted OR (95% CI) | 1.00 (ref) | **1.31 (1.06 to 1.61)** | **1.42 (1.13 to 1.79)** | **1.64 (1.27 to 2.12)** |
| **Mercury** |  |  |  |  |
| **Male** |  |  |  |  |
| MASLD ^a^ |  |  |  |  |
| Mean (SD) | 1.78 (0.47) | 3.03 (0.35) | 4.57 (0.55) | 9.45 (4.58) |
| Adjusted OR (95% CI) | 1.00 (ref) | 1.00 (0.80 to 1.25) | 1.19 (0.96 to 1.47) | **1.30 (1.06 to 1.61)** |
| MetALD ^b^ |  |  |  |  |
| Mean (SD) | 1.80 (0.45) | 3.04 (0.33) | 4.60 (0.56) | 10.40 (6.28) |
| Adjusted OR (95% CI) | 1.00 (ref) | **2.63 (1.55 to 4.45)** | **3.31 (2.01 to 5.45)** | **4.03 (2.46 to 6.60)** |
| ALD ^c^ |  |  |  |  |
| Mean (SD) | 1.79 (0.44) | 3.05 (0.35) | 4.61 (0.56) | 9.55 (7.56) |
| Adjusted OR (95% CI) | 1.00 (ref) | 1.26 (0.99 to 1.59) | **1.26 (1.01 to 1.57)** | **1.63 (1.32 to 2.03)** |
| **Female** |  |  |  |  |
| MASLD ^a^ |  |  |  |  |
| Mean (SD) | 1.80 (0.44) | 3.02 (0.37) | 4.50 (0.54) | 8.49 (3.66) |
| Adjusted OR (95% CI) | 1.00 (ref) | 1.11 (0.92 to 1.34) | **1.59 (1.32 to 1.91)** | **1.28 (1.04 to 1.57)** |
| MetALD ^b^ |  |  |  |  |
| Mean (SD) | 1.85 (0.48) | 3.04 (0.35) | 4.47 (0.56) | 7.98 (2.68) |
| Adjusted OR (95% CI) | 1.00 (ref) | **2.36 (1.43 to 3.88)** | **2.53 (1.51 to 4.23)** | **2.60 (1.48 to 4.55)** |
| ALD ^c^ |  |  |  |  |
| Mean (SD) | 1.83 (0.43) | 2.99 (0.37) | 4.53 (0.55) | 7.99 (2.67) |
| Adjusted OR (95% CI) | 1.00 (ref) | 1.09 (0.88 to 1.34) | 0.95 (0.75 to 1.19) | **1.39 (1.09 to 1.78)** |
| **Cadmium** |  |  |  |  |
| **Male** |  |  |  |  |
| MASLD ^a^ |  |  |  |  |
| Mean (SD) | 0.46 (0.15) | 0.84 (0.10) | 1.25 (0.13) | 2.07 (0.49) |
| Adjusted OR (95% CI) | 1.00 (ref) | 1.04 (0.87 to 1.26) | 1.03 (0.85 to 1.26) | 0.95 (0.77 to 1.17) |
| MetALD ^b^ |  |  |  |  |
| Mean (SD) | 0.46 (0.15) | 0.85 (0.10) | 1.21 (0.12) | 2.07 (0.64) |
| Adjusted OR (95% CI) | 1.00 (ref) | **1.82 (1.36 to 2.44)** | **1.90 (1.39 to 2.60)** | **1.59 (1.11 to 2.27)** |
| ALD ^c^ |  |  |  |  |
| Mean (SD) | 0.49 (0.14) | 0.86 (0.10) | 1.25 (0.13) | 2.07 (0.68) |
| Adjusted OR (95% CI) | 1.00 (ref) | 1.19 (0.99 to 1.43) | **1.45 (1.20 to 1.75)** | **1.52 (1.24 to 1.85)** |
| **Female** |  |  |  |  |
| MASLD ^a^ |  |  |  |  |
| Mean (SD) | 0.51 (0.13) | 0.86 (0.10) | 1.25 (0.13) | 2.16 (0.68) |
| Adjusted OR (95% CI) | 1.00 (ref) | 0.94 (0.76 to 1.17) | 1.01 (0.80 to 1.27) | 0.97 (0.77 to 1.21) |
| MetALD ^b^ |  |  |  |  |
| Mean (SD) | 0.47 (0.13) | 0.86 (0.12) | 1.25 (0.15) | 2.22 (0.73) |
| Adjusted OR (95% CI) | 1.00 (ref) | 0.80 (0.45 to 1.42) | 0.75 (0.43 to 1.32) | 0.85 (0.49 to 1.46) |
| ALD ^c^ |  |  |  |  |
| Mean (SD) | 0.48 (0.14) | 0.85 (0.10) | 1.25 (0.14) | 2.13 (0.72) |
| Adjusted OR (95% CI) | 1.00 (ref) | 1.08 (0.85 to 1.38) | 1.20 (0.93 to 1.55) | 1.21 (0.93 to 1.58) |

Abbreviations: SD, standard deviation; OR, odds ratio; CI, confidence interval; MASLD, metabolic dysfunction-associated steatotic liver disease; MetALD, metabolic alcohol-related liver disease; ALD, alcohol-related liver disease.

The figures in bold indicate statistical significance (p-value < 0.05).

^a^ The logistic regression model was adjusted for sex (male and female), age, education level (elementary school or less, middle school, high school, college or more, and unknown), household income (lowest, second, third, highest quartile, and unknown), smoking status (non-smoker, ex-smoker, current smoker and unknown) and total caloric intake (lowest, second, third, highest quartile, and unknown).

^b^ The logistic regression model was adjusted for sex, age, region of residence, education level, household income, smoking status and total number of kcal.

^c^ The logistic regression model was adjusted for sex, age, household income, smoking status and total number of kcal.
