# Supplementary material for: Association between blood heavy metal levels and subtypes of steatotic liver disease: A nationally representative cross-sectional analysis in South Korea
Source: Medicine (Baltimore). 2026 Jan 23;105(4):e47365. doi: 10.1097/MD.0000000000047365 (PMC12851683; doi:10.1097/MD.0000000000047365)
Supplement: Supplementary file 2 [file medi-105-e47365-s002.docx]

**Figure S1.** Study population flowchart**.**
